# Effects of acute alcohol consumption on neuronal activity and cerebral vasomotor response

**DOI:** 10.1007/s10072-021-05273-4

**Published:** 2021-04-30

**Authors:** Eszter Balogh, Tamás Árokszállási, Katalin Körtefái, Veronika Éva Nagy, László Csiba, László Oláh

**Affiliations:** 1 Department of Neurology, Faculty of Medicine, University of Debrecen, Móricz Zsigmond str. 22., Debrecen, H-4032 Hungary; 2 Cerebrovascular and Neurodegenerative Research Group, Hungarian Academy of Sciences, University of Debrecen, Debrecen, Hungary

**Keywords:** Acute alcohol consumption, Cerebral vasoreactivity, Neurovascular coupling, Transcranial Doppler, Visual activation

## Abstract

**Introduction:**

In the majority of European countries, driving after drinking small-moderate amount of alcohol is legal. Motivated by our previous studies on cerebral hemodynamics, we aimed to study whether a small-moderate blood alcohol content (BAC), at which driving is legal in some countries (0.8 g/L), influences the neuronal activity, neurovascular coupling, and cerebral vasoreactivity.

**Methods:**

Analyses of pattern-reversal visual evoked potential (VEP) and transcranial Doppler (TCD) examinations were performed in thirty young healthy adults before and 30 min after alcohol consumption. Cerebral vasoreactivity was evaluated by breath holding test in both middle cerebral arteries. By using a visual cortex stimulation paradigm, visually evoked flow velocity response during reading was measured in both posterior cerebral arteries (PCA).

**Results:**

The BAC was 0.82 g/L and 0.94 g/L 30 and 60 min after drinking alcohol, respectively. Latency of the VEP P100 wave increased after alcohol consumption. Resting absolute flow velocity values increased, whereas pulsatility indices in the PCA decreased after alcohol ingestion, indicating vasodilation of cerebral microvessels. Breath holding index and the visually evoked maximum relative flow velocity increase in the PCA and steepness of rise of the flow velocity curve were smaller after than before alcohol consumption.

**Conclusion:**

BAC close to a legal value at which driving is allowed in some European countries inhibited the neuronal activity and resulted in dilation of cerebral arterioles. Cerebral vasodilation may explain the decrease of cerebral vasoreactivity and might contribute to the disturbance of visually evoked flow response after alcohol consumption.

## Introduction

Except for some Central European Countries, driving after alcohol consumption is legal: in most of the European countries, the blood alcohol content (BAC) drink driving limit is 0.5 gram per litre (g/L); moreover, in the UK and Malta, the limit is 0.8 g/L [1]. The statement that drivers under the influence of alcohol have higher accidental risk is uncontroversial. The increased risk can mostly be explained by reduced attentional and cognitive capacities and delay in taking actions [2]. The background of these effects has been in the focus of numerous studies for decades. Motivated by our previous studies on cerebral hemodynamics, we intended to study whether a small-moderate blood alcohol content, at which driving is legal in some countries (0.8 g/L), influences the neuronal activity and cerebral circulation.

Regarding the effects of alcohol consumption on neuronal activity, depressive effects and delayed nerve conduction are well known [3]. Moderate dose of alcohol was shown to cause deterioration of cognitive and motor performances, reduction of whole brain metabolism [4], and decrease of the visually evoked occipital cortex activation [5]. Concerning the physiological effects of ethanol on cerebral blood flow, high dose of ethanol (BAC > 2 g/L) causes vasoconstriction [6, 7]; however, lower doses are either vasoconstrictor [6], vasodilator [8], or ineffective [7] on the cerebral arterioles of animal brain. The clue for this contradiction seems to be the metabolism of ethanol: whereas direct ethanol is vasoconstrictor, the main metabolite acetaldehyde and acetate have vasodilator effects, and in the human brain, these metabolites can dominate only at low to moderate ethanol doses depending on the capacity of alcohol dehydrogenase [8, 9]. These findings are supported by the results of human arterial spin labelling magnetic resonance imaging examinations showing that moderate doses of alcohol increase the global brain perfusion and cerebral blood flow [10, 11].

Regarding the complex effects of acute ethanol consumption on neuronal activity and cerebral blood flow, some authors suggested the direct effect of alcohol on cerebral circulation [5, 12–14]. Although several studies aimed to determine the cerebrovascular effects of acute alcohol consumption, the effects of alcohol on cerebral blood flow regulation, including the cerebral vasoreactivity and the neurovascular coupling, have not been investigated yet. As alcohol may influence both the neuronal activity and cerebral blood flow, simultaneous measurement of neuronal activation and cerebral blood flow parameters is essential to draw proper conclusions regarding the effects of alcohol on cerebral blood flow regulation.

In order to investigate the complex effect of ethanol on neuronal activity and cerebral circulation, visual evoked potential examination, flow velocity measurement in response to breath holding, and visually-evoked flow velocity response were investigated before and 30 min after alcohol consumption (target BAC 0.8 g/L).

## Material and methods

Thirty young healthy adults (15 males and 15 females) between 21 and 28 years of age (mean age 24.1 ± 1.6 years) were included in the study. The study was approved by the local ethics committee and the Office of the Chief Medical Officer. Each volunteer gave written informed consent. Subjects with cerebrovascular risk factors such as arterial hypertension, extreme obesity (body mass index higher than 35 kg/m^2^), diabetes mellitus, and hyperlipidaemia, as well as alcohol dependency, history of migraine, coronary, or peripheral artery diseases, were excluded. The study protocol included a complete neurological and ophthalmological examination, carotid and vertebral artery duplex, and routine clinical laboratory tests (serum ions, creatinine, fasting glucose, hepatic enzymes, creatinine-kinase, serum lipids, and inflammatory markers). Blood was drawn after an overnight fast between 7 and 9 a.m.

### Functional TCD study

Two 2 MHz probes were mounted by an individually fitted headband over the temporal cranial window. For measurement of vasoreactivity, the M1 segment of the MCA was insonated bilaterally at a depth of 50 mm. For evaluation of neurovascular coupling, the P2 segment of the PCA was insonated on both sides at a depth of 58–60 mm. Peak-systolic (PSV) and time averaged mean (TAMV) blood flow velocities as well as pulsatility indices (PI) were recorded with a Multidop T Doppler device (DWL, Singen, Germany). The procedure of finding and identifying the vessels followed the description of Fujioka and Donville [15] for the transtemporal approach. Since the effect of a vasodilator stimulus is more pronounced on the mean flow velocity values, but the peak-systolic flow velocities are less influenced by Doppler artefacts [16], both the mean and the peak-systolic velocity indices were used for the analysis.

Cerebral vasoreactivity was investigated by analysing the effect of breath holding on the flow velocity increase (BHI) in both MCAs. At the end of a deep inspiration, subjects were asked to hold their breath for a period of 40 s. Systolic and mean blood flow velocities in the MCAs were recorded before (baseline), during, and after the breath holding period. Maximum flow velocity value measured within 10 s after the breath holding period was used for the analysis. BHI was calculated by dividing the percent increase in mean flow velocity by the duration of breath-holding period (40 s) [17].

**Fig. 1:**
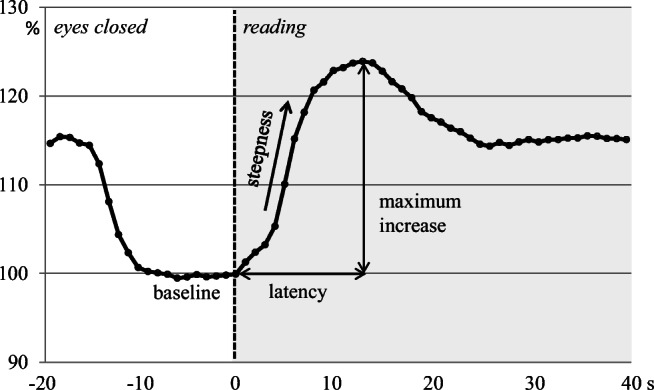
Schematic figure of the visually evoked flow response in the posterior cerebral artery. With a short time delay at the beginning of the visual stimulation (0 s), cerebral blood flow velocity increased rapidly, overshot, and then stabilized at a constant but higher level than the baseline. The analysed parameters were the maximum flow velocity increase, the latency of the maximum increase, and the steepness of the increasing slope

Neurovascular coupling was evaluated by using a visual cortex stimulation paradigm. Visually evoked flow velocity response (VEF) during reading was measured in both PCAs. As a stimulation paradigm, we used an emotionally neutral text that the volunteers could read freely. The stimulation protocol consisted of 10 cycles with a resting phase of 20 s and a stimulation phase of 40 s for each cycle. In the resting phases, the subjects closed their eyes; in the stimulation phases, they were reading. Beat-to-beat intervals of cerebral blood flow velocity data were interpolated linearly with a “virtual” time resolution of 10 ms for averaging procedures. Within one person, flow velocity data of 10 cycles were averaged. To ensure independence from the insonation angle and to allow comparisons between volunteers, absolute data were transformed into relative changes of cerebral blood flow velocity in relation to baseline. Baseline was calculated from the blood flow velocity averaged for a time span of 5 s at the end of the resting phase, before the beginning of the stimulation phase. With a short time delay at the beginning of the visual stimulation, cerebral blood flow velocity increased rapidly, overshot, and then stabilized at a constant but higher level than the baseline (Fig. 1). To analyse the maximum increase of relative flow velocity changes, the highest of the relative values obtained during the stimulation phase was taken from each subject. Additional parameters such as latency (time elapsed from stimulus onset until the maximum increase), and steepness of the increasing flow velocity curve, were also calculated (Fig. 1). Relative flow velocities were expressed in percentage of baseline.

### Experimental design: study protocol

**Fig. 2:**
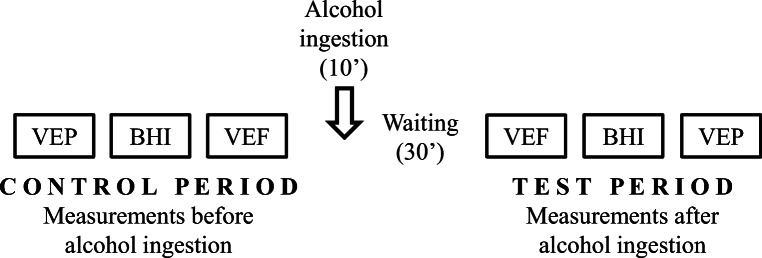
Experimental protocol. The experimental protocol in the control period included the examination of visual evoked potential (VEP), breath holding index (BHI), and visually evoked flow velocity (VEF). After the control measurements, alcohol was administered orally over a 10-min period. Following an additional 30-min pause, the same protocol was performed in the test period in a reversed order (VEF, BHI, VEP examination)

Every examination was performed before (control period) and after (test period) drinking alcohol (Fig. 2). First, visual evoked potentials by checkerboard pattern reversal stimulation were recorded over the occipital cortex (Neuron-Spectrum-4/EPM, Neurosoft, Ivanovo, Russia), and latencies and amplitudes of the P100 waves were calculated. Afterward, cerebral vasoreactivity was investigated by analysing the effect of 40 s breath holding on the mean flow velocity increase (BHI) in both MCAs. Then VEF during reading was measured by transcranial Doppler (TCD) in both PCAs.

After the first set of experiments (control period), alcohol (vodka, 37.5% alcohol content) was administered orally over a 10-min period. Volunteers were allowed to dilute the alcoholic beverage with sugar-, and caffeine-free non-carbonated soft drinks up to a total volume of 200 mL. Our aim was to investigate the effects of mild-moderate drunkenness on cerebral circulation; therefore, the target BAC was 0.8 g/L. In order to reach this concentration with low variance, we used the following formula for calculating the required amount of alcohol in grams: BAC × BW × WF, where BAC means the target blood alcohol concentration expressed in g/L, BW is the body weight in kg, and WF is the Widmark factor that is 0.68 in males and 0.55 in females [18].

After the end of alcohol ingestion, the volunteers relaxed with closed eyes for 30 min before the test period (Fig. 2). During the post-alcohol test period, 30 and 60 min after the end of alcohol consumption, blood was drawn for the measurement of BAC. The second set of experiments (test period) was started 30 min after the end of alcohol administration. The experiments in the test period were performed in a reversed order compared to the control period (the VEF test was followed by the breath holding test, and eventually VEP parameters were recorded). In order to compare the absolute flow velocity values in the PCAs, the TCD probes were not removed between the VEF tests in the control and in the test period. Blood pressure and heart rate were measured noninvasively at the beginning of the experiment and in every 5 min after alcohol consumption.

### Statistical analysis

Data were expressed as means ± standard deviation (SD). Tests for normal distribution were performed, and the homogeneity of the variances was checked by an *F* test. The flow data measured on the two sides were averaged, and the averaged data were used for analysis.

Repeated measures analysis of variance (ANOVA) was applied to compare relative changes of cerebral blood flow velocities in the stimulation phases during reading before and after alcohol consumption. The results of repeated measures analysis of variance were shown by group main effect and group with time-of-measurement interaction. Group main effect showed whether there was a significant difference in flow velocities averaged over the 40-s active period during reading before and after alcohol consumption. The group with time-of-measurement interaction indicated whether the pattern of flow velocity changes over time was different in each experimental protocol. Non-significant interaction indicated that the pattern of flow velocity changes in the different experimental settings was parallel.

Blood pressure values, heart rates, VEP P100 amplitudes and latencies, and the absolute baseline flow velocity values, as well as the maximum relative flow velocity increases, the latencies, and the steepness values of the increasing slope at different periods of the experiment, were compared by paired *t*-test. A difference of *p*≤0.05 was considered statistically significant.

## Results

Data could be obtained from all volunteers, and all data were used for evaluation. Routine clinical laboratory parameters were in the reference range. Alcohol consumption resulted in an alcohol concentration of 0.82 ± 0.25 g/L measured 30 min and 0.94 ± 0.15 g/L measured 60 min after drinking alcoholic beverage. Blood pressure showed no significant changes following alcohol ingestion, while pulse rate increased significantly already 5 min after alcohol consumption and remained elevated until the end of the experiment.

### Effects of alcohol on neuronal activity: VEP parameters

All volunteers’ visual acuity was 1.0 on both sides. Parameters of VEP were within the normal range in all subjects. After alcohol ingestion, the latency of the VEP P100 wave increased (before alcohol 107.96 ± 2.40 ms vs. after alcohol 110.81 ± 3.39 ms, *p*<0.01), whereas amplitude of the VEP P100 wave decreased (before alcohol 9.69 ± 3.19 μV vs. after alcohol 8.57 ± 3.40 μV, *p*=0.01) compared to the control period.

### Effects of alcohol on cerebral vasoreactivity: breath holding index parameters

The increment of blood flow velocity in the MCAs caused by 40 s of breath holding was lower after than before alcohol consumption (before alcohol 44.09 ± 11.42 %/40s vs. after alcohol 34.88 ± 14.28 %/40s, *p*<0.01).

### Effects of alcohol on neurovascular coupling: visually evoked flow parameters

**Fig. 3:**
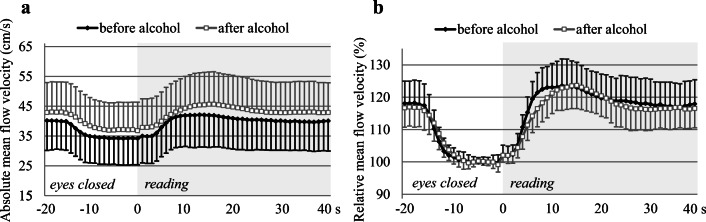
Absolute (**a**) and relative (**b**) mean flow velocity (TAMV) time courses during reading measured in the PCAs before and after alcohol consumption. SD bars are upward in Fig. 3a on the curve “after alcohol” and in Fig. 3b on the curve “before alcohol,” while they are downward in Fig. 3a on the curve “before alcohol” and in Fig. 3b on the curve “after alcohol.” Note that the absolute flow velocity was higher after than before alcohol consumption (Fig. 3a). The increase of flow velocity was much slower after than before alcohol consumption (Fig. 3a and b)

At first, baseline absolute flow velocity parameters measured in the PCAs before and after alcohol ingestion were compared. Blood flow velocities recorded for a time span of 5 s at the end of the resting phase were considered as baseline. Higher baseline absolute PSV (before alcohol 53.18 ± 12.80 cm/s vs. after alcohol 55.75 ± 12.98 cm/s, *p*<0.01) and TAMV (before alcohol 34.54 ± 9.34 cm/s vs. after alcohol 37.42 ± 9.79 cm/s, *p*<0.01; Fig. 3a), and lower PI (before alcohol 0.85 ± 0.14 vs. after alcohol 0.76 ± 0.14, *p*<0.01) were registered in the PCAs after than before alcohol consumption).

Then relative flow velocity values measured during the visual stimulation before and after alcohol ingestion were calculated in relation to the proper baseline values. The visually evoked relative flow velocity time courses between the control and test periods did not show significant group main effect (*p*=0.85 for the PSV and *p*=0.29 for the TAMV values). These data indicate that the relative flow velocities during visual stimulation were not significantly different before and after alcohol consumption. The group with time of measurement interaction, however, was significant in both peak-systolic and mean flow velocity values (*p*<0.01), which means that the pattern of flow velocity changes was different before and after alcohol ingestion (Fig. 3b). Analysis of different parameters of the relative flow velocity time courses showed that the maximum increase of relative flow velocity of TAMV values was lower, the latency of both the PSV and TAMV values was longer, and the steepness of the increasing slope of both the PSV and TAMV values was smaller after than before alcohol consumption (Table 1).

**Table 1:** Analysis of maximum relative flow velocity increase, latency, and steepness of rise of the flow velocity curve (mean ± SD) measured during the stimulation phase (reading) before and after alcohol ingestion, using paired *t*-test

Flow velocity (PCA)		PSV	TAMV
Maximum increase (%)	Before alcohol	119.23 ± 5.91	127.30 ± 7.60
	After alcohol	119.51 ± 6.37	125.58 ± 7.07
	*p*	0.65	0.02
Latency (s)	Before alcohol	13.53 ± 2.90	12.94 ± 3.62
	After alcohol	14.89 ± 2.84	14.66 ± 2.65
	*p*	<0.01	<0.01
Steepness of rise of the flow velocity curve (%/s)	Before alcohol	2.98 ± 0.99	4.73 ± 1.42
	After alcohol	2.37 ± 0.94	3.24 ± 1.03
	*p*	<0.01	<0.01

*PSV* peak-systolic flow velocity, *TAMV* mean flow velocity, *PCA* posterior cerebral artery, *before alcohol* before alcohol ingestion, *after alcohol* after alcohol ingestion

**Fig. 4:**
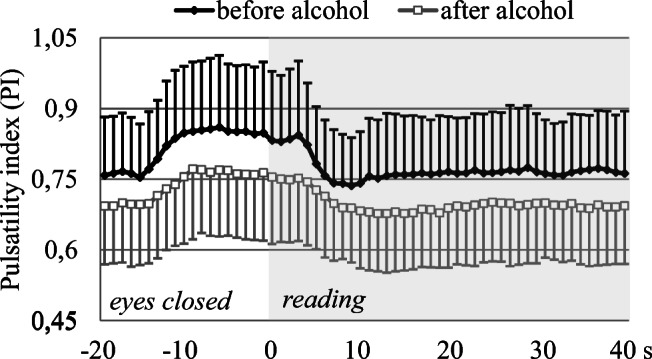
Pulsatility index (PI) time courses during reading measured in the PCAs before and after alcohol consumption. SD bars are upward on the curve “before alcohol,” while they are downward on the curve “after alcohol.” Note the lower PI after alcohol ingestion, suggesting decrease in vascular resistance. Further decrease of PI induced by visual stimulation indicates additional vasodilation of cerebral microvessels during reading not only before, but also after alcohol consumption

After alcohol consumption, the pulsatility indices were significantly lower not only in the resting phase but also during the stimulation phase compared to the corresponding values in the control period (Fig. 4). Repeated measures ANOVA revealed that both the group main effect (*p*=0.03) and the group with time-of-measurement interaction (*p*<0.01) were significant.

## Discussion

Our purpose was to study the impact of small-moderate dose of ethanol on neuronal activity and cerebral hemodynamics, including cerebral vasoreactivity and neurovascular coupling. To the best of our knowledge, cerebral vasoreactivity induced by breath holding and neurovascular coupling evoked by reading before and after alcohol consumption have never been compared.

The target 0.8 g/L BAC was reached; the concentration measured 1 h after alcohol consumption slightly exceeded the planned level. As this value is close to the BAC driving limit that applies in some European countries, our data represent the potential pathophysiological effects of this alcohol level.

Heterogeneous effects of low-to-moderate doses of ethanol on cardiovascular parameters were described previously. Studies in humans showed that acute alcohol consumption increased [19], did not affect [20], or decreased [21] the blood pressure. Most of the experiments revealed that alcohol increased the pulse rate [19, 20]. Our results are in agreement with these observations: the blood pressure showed no significant changes, while the pulse rate elevated significantly following alcohol ingestion in our study.

Consistent with depressive effects of ethanol on central nervous system, we observed significant increase in the latency and decrease in the amplitude of VEP P100 wave under the effect of alcohol. In line with our findings, other authors [22, 23] pointed out earlier that alcohol ingestion was associated with prolongation of P100 wave latency. Moderate dose of alcohol consumption was reported to reduce the whole brain metabolism, which decrease was the most pronounced in the occipital cortex [4] and may explain the changes in the VEP parameters.

Increase of baseline absolute flow velocities and decrease of the pulsatility index in the PCA after drinking alcohol suggest decreased cerebrovascular resistance indicating alcohol induced vasodilation in the cerebral resistance vessels. Similar changes in the MCA were already described after alcohol consumption [14]. Since negative effects of alcohol was found on neuronal activity, alcohol induced increase in cerebral metabolism can be excluded in the background of vasodilation. Quite contrary, our data indicate the direct effect of ethanol on the cerebral vasculature which is congruent with the results of previous studies [6, 12–14].

Our results indicated that breath holding index, that is the hypercapnia-induced vasomotor response, decreased after ethanol consumption. The physiology of cerebral vasoreactivity is that hypercapnia induces vasodilation in the resistance vessels, leading to increase in flow and flow velocities in the supplying artery [24]. The decrease in cerebral vasomotor response after alcohol consumption is probably due to the alcohol-induced dilation of cerebral microvessels. As alcohol caused a significant dilation of the cerebral resistance vessels, further vasodilator stimulus (hypercapnia) could only result in a smaller vasodilation, leading to lower breath holding evoked flow velocity response.

Although significant vasodilation developed in the territory of the PCA after alcohol ingestion, additional increase of flow velocities and decrease of pulsatility indices were observed during visual stimulation (Figs. 3 and 4), suggesting further decrement of vascular resistance in the arterioles due to the visual stimulation-induced dilation of cerebral microvessels (i.e. the neurovascular coupling).

The neurovascular coupling as a complex process was affected by alcohol consumption. Although the group main effect of relative flow velocity changes during visual stimulation did not reveal significant difference before and after ethanol ingestion, the pattern of flow velocity changes was different. Moreover, the maximum increase of relative flow velocity was lower, the latency of reaching the maximum flow velocity value was longer, and the steepness of the increasing slope was smaller after than before alcohol consumption, indicating negative effects of ethanol on neurovascular coupling. Since regional cerebral blood flow changes are coupled with regional brain activation, disturbance of neurovascular coupling could be due to the reduced neuronal activity indicated by prolonged latency and decreased amplitude of VEP P100 wave observed after alcohol consumption. Further mechanism in the background of the smaller visually evoked flow changes could be the alcohol induced dilation of cerebral microvessels, which was indicated by an increase in absolute flow velocity values and a decrease in pulsatility indices after alcohol ingestion. This cerebral vasodilation could interfere with the further dilation of the arterioles that is required for the neuronal activation induced flow response.

## Conclusions

Our data prove that acute alcohol consumption inhibits the visually evoked occipital cortex activation, and results in dilation of cerebral arterioles. The alcohol-induced cerebral vasodilation may explain the decreased cerebral vasomotor reactivity and together with the decreased neuronal activity may contribute to the compromised neurovascular coupling. Our results confirm the deteriorative effects of low-to-moderate doses of ethanol on neuronal activity and cerebral hemodynamics and support efforts to reduce the BAC drink-driving limit in certain European countries.

## Data Availability

Not applicable.
